# Spatial Control over Catalyst Positioning for Increased Micromotor Efficiency

**DOI:** 10.3390/gels9020164

**Published:** 2023-02-18

**Authors:** Shauni Keller, Serena P. Teora, Arif Keskin, Luuk J. C. Daris, Norman A. P. E. Samuels, Moussa Boujemaa, Daniela A. Wilson

**Affiliations:** Department of Systems Chemistry, Institute of Molecules and Materials, Radboud University, Heyendaalseweg 135, 6525 AJ Nijmegen, The Netherlands

**Keywords:** micromotor, microfluidics, aqueous phase separation, autonomous motion, spatial localization

## Abstract

Motion is influenced by many different aspects of a micromotor’s design, such as shape, roughness and the type of materials used. When designing a motor, asymmetry is the main requirement to take into account, either in shape or in catalyst distribution. It influences both speed and directionality since it dictates the location of propulsion force. Here, we combine asymmetry in shape and asymmetry in catalyst distribution to study the motion of soft micromotors. A microfluidic method is utilized to generate aqueous double emulsions, which upon UV-exposure form asymmetric microgels. Taking advantage of the flexibility of this method, we fabricated micromotors with homogeneous catalyst distribution throughout the microbead and micromotors with different degrees of catalyst localization within the active site. Spatial control over catalyst positioning is advantageous since less enzyme is needed for the same propulsion speed as the homogeneous system and it provides further confinement and compartmentalization of the catalyst. This proof-of-concept of our new design will make the use of enzymes as driving forces for motors more accessible, as well as providing a new route for compartmentalizing enzymes at interfaces without the need for catalyst-specific functionalization.

## 1. Introduction

New designs for micro- and nanomotors are often proposed; however, a thorough understanding of the mechanism of motion and how to influence it is still lacking. One example of this is the addition of surfactant to bubble propelled motors, which is often not clearly mentioned and discussed. This enhances bubble formation and detachment and thus increases speed [1,2]; however, it is not always noted that this changes the normal mechanism of motion. Surfactant is such a strong additive that it dominates the mechanism of motion and can hide smaller, more interesting aspects of the micromotor’s behaviour. Surfactants are also not present in biological systems, and therefore their addition in the design of motors for biomedical applications is undesirable and does not reflect real situations in biological environments. In general, the goal seems to be to design faster motors either by changing the geometric design or the surrounding environment. This makes it difficult to determine the key concepts for efficient propulsion, not necessarily fast propulsion, and their influence on motion.

Environmental factors that influence speed and motion trajectories include surfactants, surface tension and viscosity [1,3]. By adding surfactants, increasing surface tension and decreasing viscosity, the velocity of micromotors can be increased. In situ changes of the surface tension can induce Marangoni flows, which can drive motors as well as affect their motion [4,5]. At the same time, these different environmental factors can change the motor’s trajectory, as well, going from linear in highly viscous media to circular at low viscosities. There are many different aspects of a motor’s design that influence motion, as well. These include roughness [6,7], shape [7,8,9,10,11], and the materials of which the motors are made [6,12,13]. One of the main requirements of designing a self-motile system is asymmetry, which affects the motor’s behaviour greatly. Asymmetry, either in shape or catalyst distribution, dictates the location of propulsion force and, thus, speed and directionality. Shape can dictate propulsion by, for example, trapping the catalyst in a cavity with only one opening, forcing the propelling force through this one outlet [14,15,16,17]. Other methods of dictating the location of propulsion force are through concave shapes [6,18], as oversaturation is more easily reached there, or by differences in surface roughness, since roughness enables bubble pinning and can thus enhance the speed of bubble propelled motors [3,7]. Asymmetry in catalyst distribution directly dictates the location of the propulsion force, since the location of the catalyst is where the reaction happens and, thus, where the propelling products are formed. Asymmetry in catalyst distribution is easily created for inorganic motors; a spherical particle becomes a motor upon sputtering or growing a patch of catalytically active material [19,20,21]. This inorganic, Janus-type motor has been studied and modelled often due to its simple design, as well as semi-cone shapes.

Nowadays, more complex motors are designed and inorganic catalyst are often replaced with their biological counterparts [17,22,23,24]. This increases biocompatibility; however, it also increases complexity, both structurally, since the particles need different functionalization to be able to immobilize enzymes, and also practically, since enzymes are more difficult to work with as they demand a very specific working environment and can degrade or inactivate over time. Furthermore, inorganic structures often have the enzyme immobilized on the surface, which can lead to decreased activity due to functionalization and direct exposure to high concentrations of fuel or other inhibiting chemicals [25,26]. Asymmetric shapes are mostly seen in soft self-assembled particles, and the methods used for fabrication allow for more flexibility in the shape and choice of materials. Motors containing asymmetry in both shape and catalyst distribution are limited [11]; mostly, soft materials are combined with inorganic catalyst [27,28] or inorganic structures with enzymes [22,24,29].

We propose a soft micromotor design that contains asymmetry in both shape and catalyst distribution, combining hydrogel and biocatalysts. Instead of continuously designing new types of micromotors, we study the effect on autonomous motion upon slight changes in the design. For this, we keep the shape and building blocks the same but change the molecular weight or arrangement of the materials. A microfluidic chip generating aqueous-two-phase-separated droplets is utilized to achieve asymmetry in the shape of the microbeads. Due to the flexibility of this method, an easy change of solutions and catalysts is possible without altering the overall structure of the motor. By taking advantage of this, asymmetry in catalyst distribution is easily obtained for enzymes without the need for complex immobilization methods. Here, we obtain spatial control over catalyst localization to study its influence on motion.

## 2. Results and Discussion

### 2.1. Experimental Design

**Figure 1:**
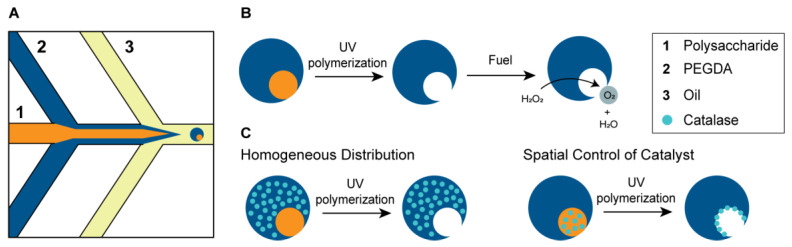
Schematic representation of the experimental design. (**A**) Close-up of the ATPS-based microfluidic chip to generate droplet-in-droplet morphology. An aqueous-two-phase jet is formed at the first cross junction, which is emulsified at the second cross junction by a surfactant containing oil. (**B**) Asymmetric microgels are obtained after UV-polymerization of the droplets generated by the microfluidic chip; upon addition to hydrogen peroxide the catalyst, catalase, will decompose the fuel to water and propelling oxygen. (**C**) Two methods to position the catalyst, either homogeneously through incorporation in the gel or spatially through adding it to the polysaccharide, templating phase.

Micromotors are made using poly(ethylene glycol) diacrylate (PEGDA) and different molecular weight polysaccharides, dextran of 10 kDa and 70 kDa and Ficoll of 400 kDa, via an aqueous-two-phase-system (ATPS) based microfluidic chip (Figure 1A). Aqueous-phase-separation is a spontaneous phenomenon based on steric exclusion of two immiscible, aqueous, polymer solutions which phase separate above a critical concentration. Our microfluidic method generates a two-phase jet of two immiscible aqueous solutions at the first cross junction, which is then emulsified by a surfactant containing oil at the second cross junction to obtain a droplet-in-droplet morphology which is collected at the outlet. This droplet-in-droplet morphology is obtained for all three polysaccharides (Figure S1, supporting information). The polysaccharide solution acts as a templating phase which, upon PEGDA crosslinking with UV-exposure, results in asymmetrical microbeads. The incorporation of a catalyst will transform these inactive microgels into autonomously moving micromotors. Here, we use a biocatalyst, catalase, which decomposes hydrogen peroxide into water and propelling oxygen (Figure 1B). The enzyme can be incorporated in either phase, PEGDA or polysaccharide, to obtain a homogeneous or asymmetric distribution, respectively (Figure 1C). The molecular weight of the polysaccharide determines whether it can diffuse into the gel or not and thereby determines the roughness inside the opening of the particle [6,30]. Polysaccharides of a molecular weight higher than 70 kDa are unable to diffuse into the gel and will thus remain inside the opening, inducing increasing roughness upon increasing molecular weight. Polysaccharides below this molecular weight are able to diffuse inside the gel and will leave a smooth opening behind them (Figure S2, supporting information). Here, we use dextran of 10 kDa and 70 kDa and Ficoll of 400 kDa, which will result in smooth, medium rough and very rough openings, respectively (Figure S3, supporting information). Catalase can be dissolved either in the PEGDA phase or in the polysaccharide phase. Dissolving the enzyme in the PEGDA phase will result in a homogeneous distribution. Upon UV-exposure, the PEGDA gel will form around the enzyme, completely enclosing it. Dissolving the enzyme in the polysaccharide phase is expected to localize the enzyme at the PEGDA-polysaccharide interface. Due to the size of the catalase, 250 kDa, it is unable to leak out of the gel when dissolved in PEGDA or diffuse completely into the gel when dissolved in the polysaccharide phase. The degree of localization will be dependent on the molecular weight polysaccharide that is used. A low molecular weight dextran diffuses into the microgel and will leave the enzyme at the PEGDA interface, while a higher molecular weight polysaccharide will not diffuse inside, resulting in a more spread distribution of the catalyst inside the opening.

### 2.2. Catalyst Distribution

**Figure 2:**
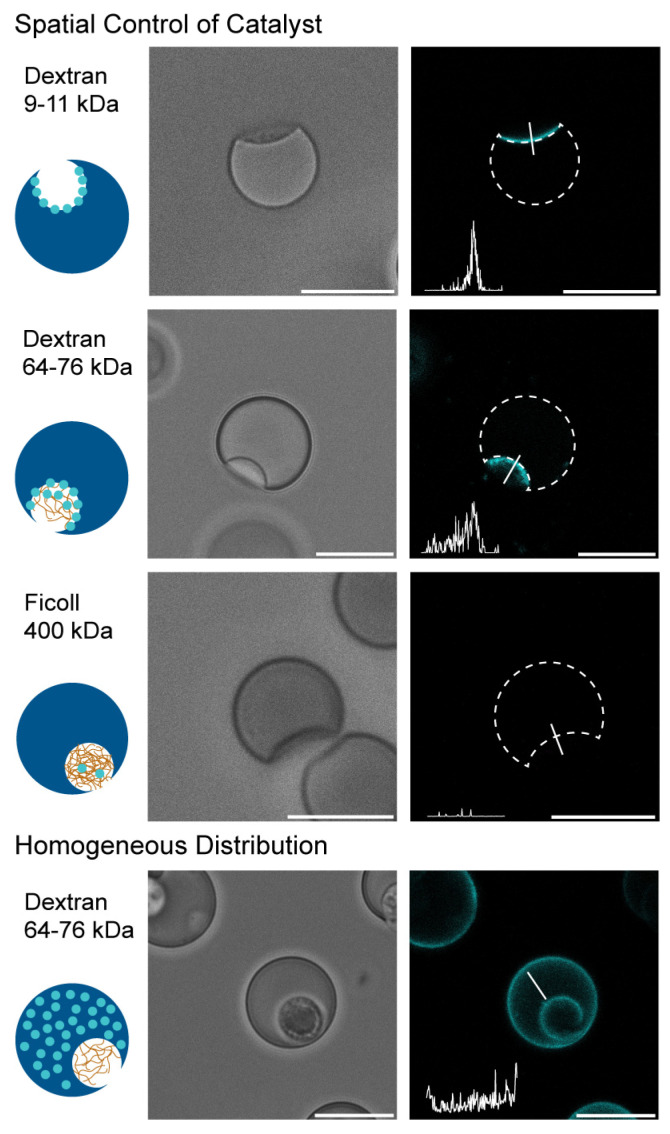
Confocal microscopy images of the different micromotor systems. Catalase was labelled with a fluorescent dye, Alexa 647, to analyse its position inside the motor. Bright field images are shown left, and the corresponding fluorescence images are on the right. The position of the motor is shown in dashed lines and the fluorescence intensity profile (bottom left) was obtained over the solid line going from the opening inside the motor. Spatial control over the catalyst was obtained by dissolving the enzyme in the polysaccharide phase prior to injection into the chip. As a control the catalyst was dissolved in the PEGDA gel phase, and a homogeneous distribution throughout the bead was observed. Scale bar is 20 µm.

Micromotors were generated containing fluorescently labelled catalase in the polysaccharide phase, and as a control fluorescently labelled catalase was dissolved in the PEGDA phase. The shape of the microgels and localization of the catalyst were studied using confocal microscopy (Figure 2). All the different microsystems were obtained in similar size and shape, as can be seen in the bright field images (Figure 2, left). Corresponding fluorescent images (Figure 2, right) show the distribution of the enzyme inside the microbeads; the position of the microgel itself is marked with a dashed line and the fluorescence intensity profile (bottom left in the fluorescence image) was obtained over the solid line going from the opening into the bead, left to right in the graph, respectively. Catalase in dextran 10 kDa is localized in a sharp line onto the inner surface of the PEGDA gel. For higher molecular weight dextran, 70 kDa, the localization is more dispersed, while there is little fluorescence visible for the Ficoll 400 kDa. As a control, fluorescently labelled catalase was dissolved in the PEGDA phase and a homogeneous distribution throughout the bead was obtained for all three polysaccharide systems (Figure 2 and Figure S4, supporting information). The observed localization is due to the (in)ability of the polysaccharide to diffuse into the gel and carry along the enzyme, depending on its molecular weight. When the PEGDA phase starts polymerizing it will contract, which enables the diffusion of low molecular weight polysaccharide into the gel. The gel will form around the polysaccharide, and thus entrap it inside. The enzyme itself has a molecular weight of 250 kDa and cannot diffuse inside the PEGDA gel; as a result, the enzyme is set off at the PEGDA inner surface. At 70 kDa dextran, the PEGDA contraction still acts on the polysaccharide phase; however, the molecular weight is too high to diffuse into the PEGDA gel and thus it remains at the PEGDA interface of the microbead. As a results, a smaller degree of enzyme diffusion is observed towards the PEGDA inner surface. Ficoll 400 kDa is more viscous because of its branched nature and therefore is not affected by the contraction of the PEGDA, and no diffusion of the polysaccharide nor the enzyme occurs at all. The enzyme will remain diffused inside the opening, and upon washing it might leak out; as a result little fluorescence is observed. Homogeneous localization of the enzyme is ensured by dissolving catalase in the PEGDA phase. Upon UV-exposure the PEGDA gel forms around the enzyme, resulting in a homogeneous distribution. The high molecular weight of the catalase inhibits the enzyme leaking out of the PEGDA gel.

### 2.3. Motion Analysis

**Figure 3:**
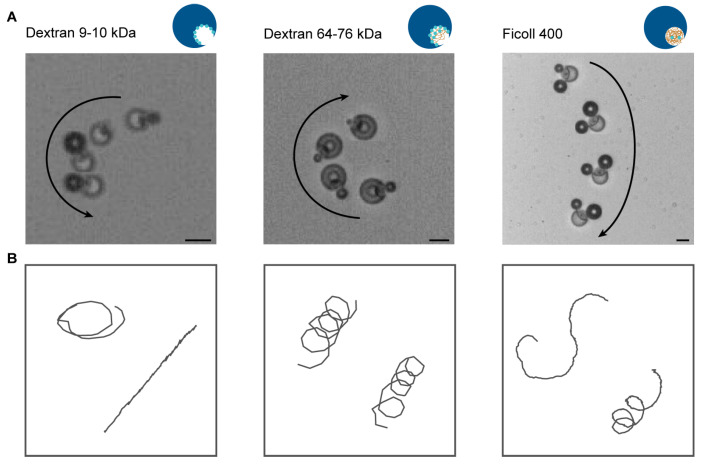
(**A**) Bright field microscopy overlay of bubble propulsion of the three different polysaccharide systems with an interval of 0.5, 1, and 6 s for dextran 10 kDa, 70 kDa and Ficoll 400 kDa, respectively. (**B**) Typical trajectories of each motor system. Scale bar is 20 µm.

By localizing the catalyst inside the opening of the motor, less catalyst is needed to propel the motor. The maximum amount of enzyme in the dextran phase is three times less than the amount of catalyst in the PEGDA phase, and this is theoretically calculated from the catalyst concentration and the volume of the droplets. For the homogeneous system, the PEGDA gel is formed around the catalase and is thus encapsulating it completely. Due to the enzyme’s high molecular weight, 250 kDa, it is maintained inside the gel and cannot leak out. For the localized system, however, diffusion is the main driving force and the enzyme is not fully incorporated inside the gel. Therefore, some of the enzyme might leak out upon the washing of the motors, such as with the Ficoll 400 kDa system. The actual amount of enzyme present in the motor is thus less and might differ in between motors and in between batches. Motors were added to 4% hydrogen peroxide solution and tracked at 20 s after addition to the fuel for 10 s. The enzyme decomposes the hydrogen peroxide into water and propelling oxygen. Oxygen bubbles are formed in the opening of the motor, as oversaturation is easily reached here due to the concave shape and catalase localization, and by the easy pinning of bubbles due to surface roughness. Oxygen bubbles nucleate inside the opening, propelling the motors in the opposite direction (Figure 3A). Circular trajectories are dominant for all three systems (Figure 3B). This is due to the location of the bubble pinning, which is more likely to be off-centre than exactly in the centre of the opening, resulting in circular motion. For dextran 10 kDa we observe linear trajectories as well, and this system relies more than the other systems on oversaturation to form bubbles. Bubbles formed by oversaturation occupy the whole opening and are more likely to result in linear trajectories. For Ficoll 400 kDa, we observe a combination of circular and tumble-and-run-like trajectories. This is due to the generation and collapse of multiple bubbles, which destabilize the strictly circular motion.

**Figure 4:**
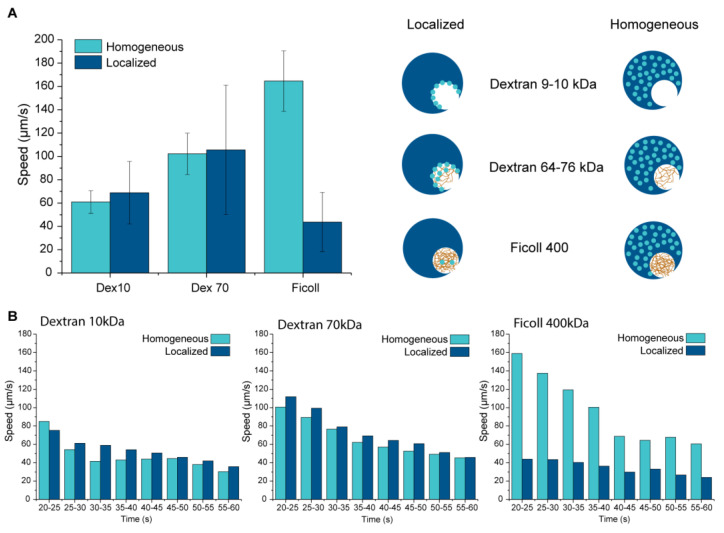
(**A**) The average speed over 10 s of the enzyme-localized systems compared to the previously obtained homogeneous systems [6] for all three polysaccharides at 4% hydrogen peroxide concentration. (**B**) The instantaneous speed of both systems was analysed over a time-course up to 60 s after addition at 4% hydrogen peroxide concentration.

Even though the catalase content is very low in the localized systems, the motors are propelled efficiently (Figure 4A). Compared to previous data with homogeneous catalyst distribution [6], localized enzymes result in similar speeds for both dextran 10 kDa and 70 kDa. Since the localized system is based on diffusion and not all of the enzyme is maintained, larger differences in speed in the same batch and between batches exist, resulting in larger error bars. The speed for Ficoll 400 kDa drastically decreased, as was expected, since little catalase was observed in this system. Even though the catalyst loading is very low, this system has the highest roughness, which makes bubble pinning easier. Therefore, bubble propelled motion is still observed.

Enzyme activity assays were conducted to study the role of UV-exposure and the polymer solution on enzyme activity (Figure S5, supporting information). This was performed by monitoring the decrease in absorbance of hydrogen peroxide at a wavelength of 240 nm over time. Catalase decomposes hydrogen peroxide, which will decrease its concentration and thus the absorbance at 240 nm wavelength. Catalase solutions before and after UV-exposure exhibit the same enzyme activities, and thus the UV-exposure used to crosslink the microbeads does not damage the enzyme. The enzyme activity in the PEGDA phase is similar for dextran 10 kDa and dextran 70 kDa solutions, showing increased activities compared to Milli-Q, while in Ficoll 400 the activity decreased. These polymers are often used as crowding and stabilizing agents, and are known to affect enzyme activities [31,32,33,34]. There is no increased enzyme activity in polysaccharide solutions compared to the PEGDA solution, and therefore this cannot be the cause of the relatively high speeds obtained by the localized system. The relatively high speeds obtained with very low enzyme loading are thus a result of spatial localization of the enzyme. This ensures a local high concentration of the enzyme, and together with its confinement inside the opening and possible stabilization by dextran the fuel can be decomposed efficiently. This also implies that, for the homogeneous system, not all of the enclosed enzyme might be contributing to bubble propulsion, only the catalase located at the microgels’ surface.

The speed of the motors over time was studied as well. This was undertaken by analysing the instantaneous speed, which is the speed for each time point (Figure 4B). The instantaneous speed of the motors was averaged for each 5 s and plotted from 20 up to 60 s after the addition of the motors to the fuel. For both dextran 10 kDa and 70 kDa localized systems, the motors seem to maintain slightly higher speeds over time than the corresponding homogeneous systems; however, the overall trend of both homogeneous and localized systems is similar. The speed for localized Ficoll 400 kDa decreases slowly, while we see a continuous rapid decrease in speed for the homogeneous system. The decrease of speed over time is due to the inhibition of the enzyme by its own fuel [25]. Even though the enzyme is more exposed to the environment in the localized system compared to the homogeneous system, the activity over time is not affected.

## 3. Conclusions

Here, we reported the proof-of-concept design to obtain spatial control over biocatalysts via a simple and general method that does not require complex functionalization. The microfluidic method shown here can be used to generate double emulsions from any ATPS couple and asymmetric microgels when one of the phases is cross linkable, and it is therefore very versatile in its use. Cargo can be easily replaced for other (bio)catalysts or nanoparticles and switched from one phase to the other. This makes it an ideal tool to study the influence of different aspects on the microparticle’s structure and the micromotor’s autonomous motion. PEGDA-polysaccharide ATPSs were used to obtain double emulsions, after which micromotors were successfully generated. Catalyst can be incorporated in either phase, PEGDA or polysaccharide, resulting in a homogeneous or localized distribution, respectively. Different molecular weight polysaccharides result in different degrees of spatial control. A sharp localization at the microparticles’ inner surface was obtained at low molecular weights, while at high molecular weights the enzyme was more diffused, but still localized inside the opening of the particle. The advantage of localizing the catalyst at the action-site is that it is more concentrated and confined, and therefore less enzyme is needed to obtain efficient motion. Even though the enzyme is more exposed to the environment at the particles interface compared to inside the gel, the motors still obtain similar speeds and the activity over time is not affected.

This new method will open up new possibilities for the compartmentalization of cargo and the use of enzymes as propelling agents. Even though enzymes are biocompatible and superior in activity and sensitivity over inorganic catalysts, they do exhibit a narrow range of optimum efficiency and have a short lifetime outside their natural environment. This makes them more difficult to handle than their inorganic counterparts and they are, therefore, unfortunately not used as much. This new design provides a simple method to obtain spatial control over the localization of biocatalysts while maintaining their activity, which will make the use of biocatalysts as propelling forces more accessible.

## 4. Materials and Methods

### 4.1. Materials and Reagents

All chemicals and enzymes were used as received unless otherwise stated. Sylgard^®^ 184 silicone elastomer kit was used for the fabrication of the PDMS microfluidic chip. Trichloro(1H,1H,2H,2H-perfluorooctyl)silane (97%), dextran from leuconostoc mesenteroides (average mol wt 9–11 kDa, and 64–76 kDa), FITC-dextran average mol wt 10,000 and 70,000 (λex 492 nm, λem 518 nm), Ficoll 400, poly(ethylene glycol) diacrylate (average Mn 575), photoinitiator 2-hydroxy-4′-(2-hydroxyethoxy)-2-methylpropiophenone (98%), 1H,1H,2H,2H-perfluoro-1-octanol (97%), and Catalase form bovine liver (>99%, >20,000 u/mg and >10,000 u/mg) were purchased from Sigma-Aldrich. Alexa Fluor^®®®^ 647 NHS Ester was purchased from ThermoFischer. FluoroSurfactant (008) was purchased from Ran Biotechnologies.

### 4.2. PDMS Microfluidic Device

Sylgard^®^ monomer and initiator (10:1 *w/w*) were mixed and poured onto the silicon master, after which it was degassed under vacuum for at least 4 h. The PDMS was cured at 65 °C overnight, washed with isopropanol and blow dried. After oxygen plasma treatment, the PDMS was bonded to a precleaned glass slide. The channels were coated with trichloro(1H,1H,2H,2H-perfluoro-octyl)silane (2% *w/w* in fluorinated oil) and the device was baked at 110 °C overnight.

### 4.3. Micromotor Fabrication

All solutions were flushed with nitrogen for at least 0.5 h, and the fluorocarbon oil (HFE 7500) was flushed for 15 min, to remove dissolved oxygen. Dextran (20% *w/w*) and PEGDA (40% *w/w*) were independently injected in the first cross-junction. The photo-initiator was added (0.4 wt% final concentration) to the PEGDA solution prior to injection. The droplets were formed at the second cross-junction by the introduction of an outer phase which consisted of a fluorocarbon oil (HFE 7500) and a surfactant (SS08, 2% *w/w*). The resulting emulsion was collected in an Eppendorf. UV curing of PEGDA was achieved by exposing the emulsion to a focused UV beam (λ = 300–600 nm, 5 min, 20% intensity). The emulsion was broken by adding 1H,1H,2H,2H-Perfluoro-1-octanol (100μL, 20% *w/w* in hexane), after which the beads were washed rigorously with MiliQ to ensure that no surfactant remained.

Catalase (>20,000 u/mg) was coupled with Alexa Fluor^®®®^ 47 dye as described in the manual and was added prior to injection, with 6 mg/mL and 18 mg/mL for the PEGDA and polysaccharide phase, respectively. The volume ratio of PEGDA:polysaccharide is roughly 9:1, taking into account the relative concentrations, and the overall catalase content is three times higher in the homogeneous system compared to the localized system. Flowrates: Oil: 600 µL/h, PEGDA: 60 µL/h, Dextran 10 kDa: 20 µL/h, Dextran 70 kDa and Ficoll 400 kDa: 10 µL/h.

### 4.4. Activity Assay

The activity assay of the catalase was performed by monitoring the absorbance of hydrogen peroxide at a wavelength of 240 nm over time. Catalase solutions were made with the aforementioned polymer concentrations and diluted to a final enzyme concentration of 400 u/mL. To the cuvette was added Milli-Q (890 µL), H_2_O_2_ (0.35%, 100 µL), and enzyme solution (400 u/mL, 10 µL). Time-spectra were taken, measuring absorption every 5 s.

### 4.5. Autonomous Movement Experiments

A Petri dish of 3 cm in diameter was filled with 4% hydrogen peroxide solution (3 mL), after which the micromotor suspension (2 µL) was added. Recording started upon addition of the micromotors to the fuel solution. Afterwards, the movies were analysed using a custom-build tracking software. Tracking started 20 s after addition for a period of 10 s and the average speed was calculated over the ten fastest micromotors of each movie and averaged over three different batches.

## Data Availability

Data are contained within the article or supplementary material.

## Supplementary Materials

The following supporting information can be downloaded at: https://www.mdpi.com/article/10.3390/gels9020164/s1, Figure S1. Bright field images of the droplet-in-droplet morphology of all three polysaccharide system directly obtained from the microfluidic set-up. The large phase (large circle) is the PEGDA phase, while the small phase (small circle) is the polysaccharide phase. Scale bar is 20 µm. Figure S2. Confocal images of FITC-labelled dextran in corresponding molecular weight of 10 kDa and 70 kDa. Figure S1. Cryo-scanning electron microscopy images of all three polysaccharide systems with smooth (left, dextran 9–11 kDa), medium rough (middle, dextran 64–76 kDa) and very rough (right, Ficoll 400 kDa) openings. Scale bar is 10 µm. Figure S4. Confocal images of the fluorescently labelled catalase dissolved in the PEGDA phase. For all three polysaccharide systems a homogeneous distribution is obtained, as was expected. Scale bar is 20 µm. Figure S5. Activity assay of catalase before and after UV-exposure (left) and in different aqueous solutions (right).
