# Supplementary material for: Spatial Control over Catalyst Positioning for Increased Micromotor Efficiency
Source: Gels. 2023 Feb 18;9(2):164. doi: 10.3390/gels9020164 (PMC9957166; doi:10.3390/gels9020164)
Supplement: Supplementary file 1 [file gels-09-00164-s001.zip › gels-2222220-supplementary.pdf]

*Supporting Information*

# Spatial Control over Catalyst Positioning for Increased Micromotor Efficiency

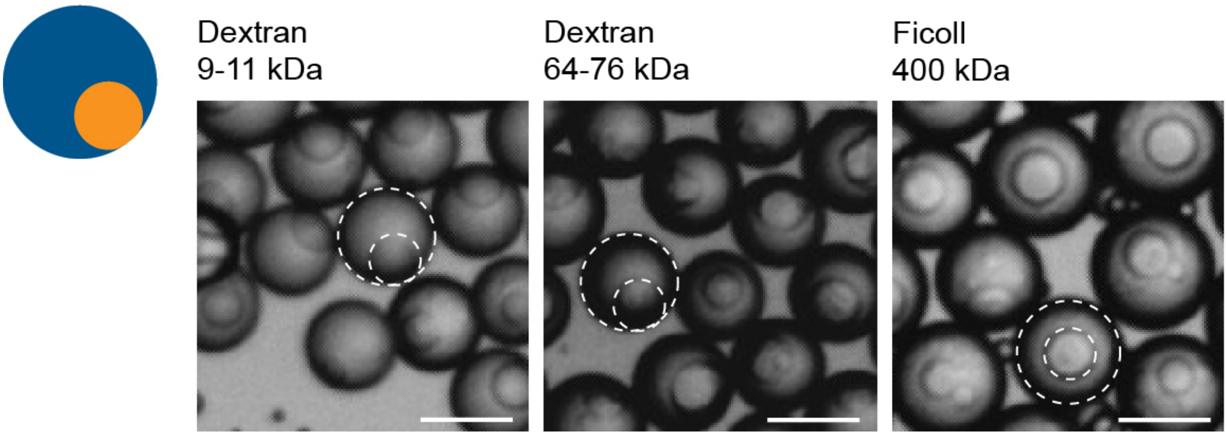


**Figure S1.** Bright field images of the droplet-in-droplet morphology of all three polysaccharide system directly obtained from the microfluidic set-up. The large phase (large circle) is the PEGDA phase, while the small phase (small circle) is the polysaccharide phase. Scale bar is 20 µm.

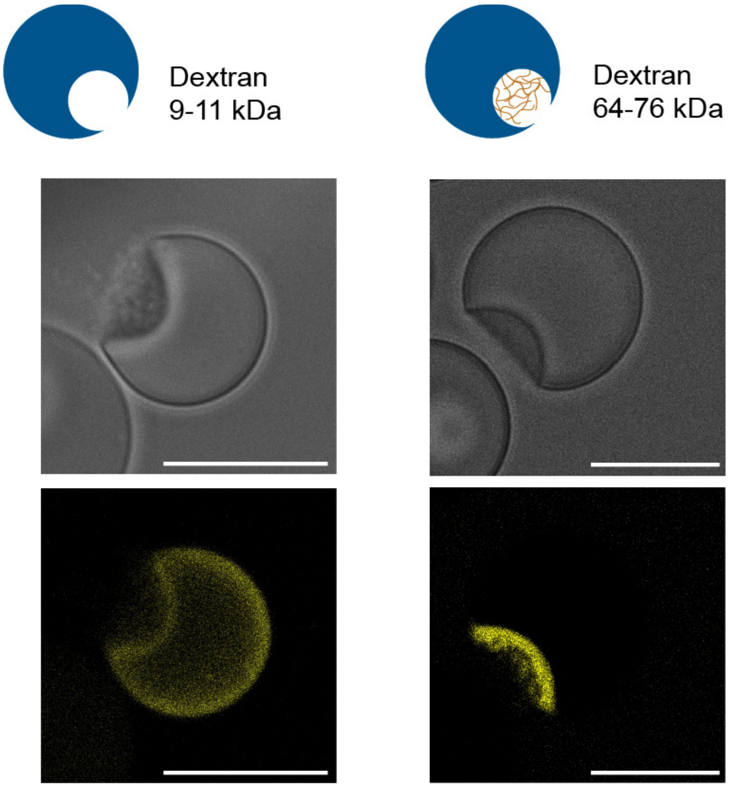


**Figure S2.** Confocal images of FITC-labelled dextran in corresponding molecular weight of 10 kDa and 70 kDa. Dextran of a molecular weight of 10 kDa is able to diffuse inside the PEGDA gel upon crosslinking resulting in homogeneous distribution of dextran throughout the gel and a clean socket. Dextran of 70 kDa molecular weight is too large to diffuse inside the gel and remains inside the opening after crosslinking. Scale bar is 20 µm.

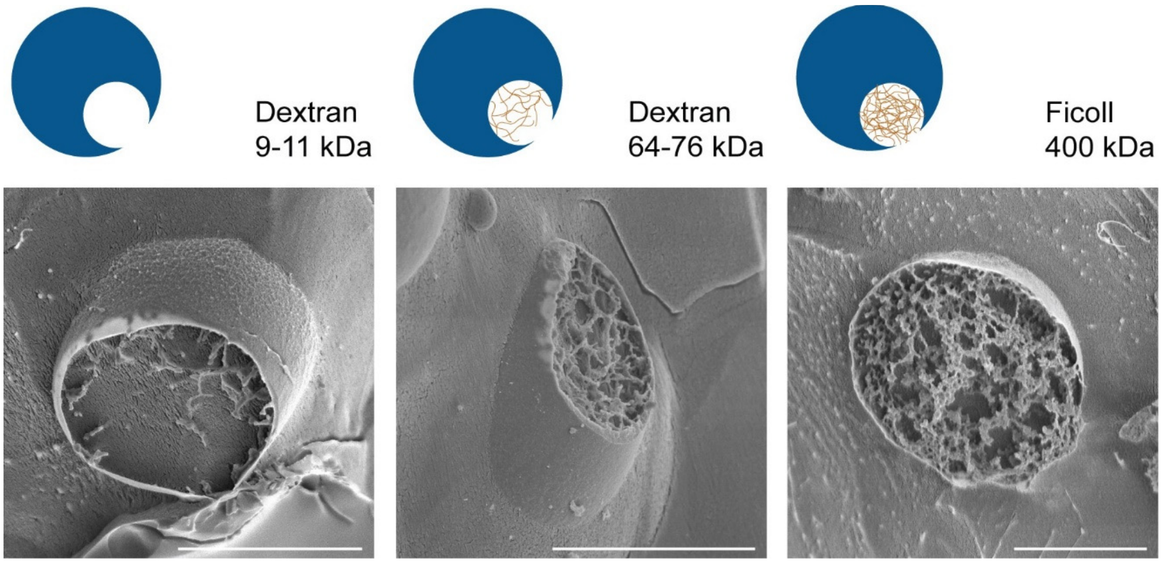


**Figure S3.** Cryo-scanning electron microscopy images of all three polysaccharide systems with smooth (left, dextran 9-11 kDa), medium rough (middle, dextran 64-76 kDa) and very rough (right, Ficoll 400 kDa) openings. Scale bar is 10 μm.

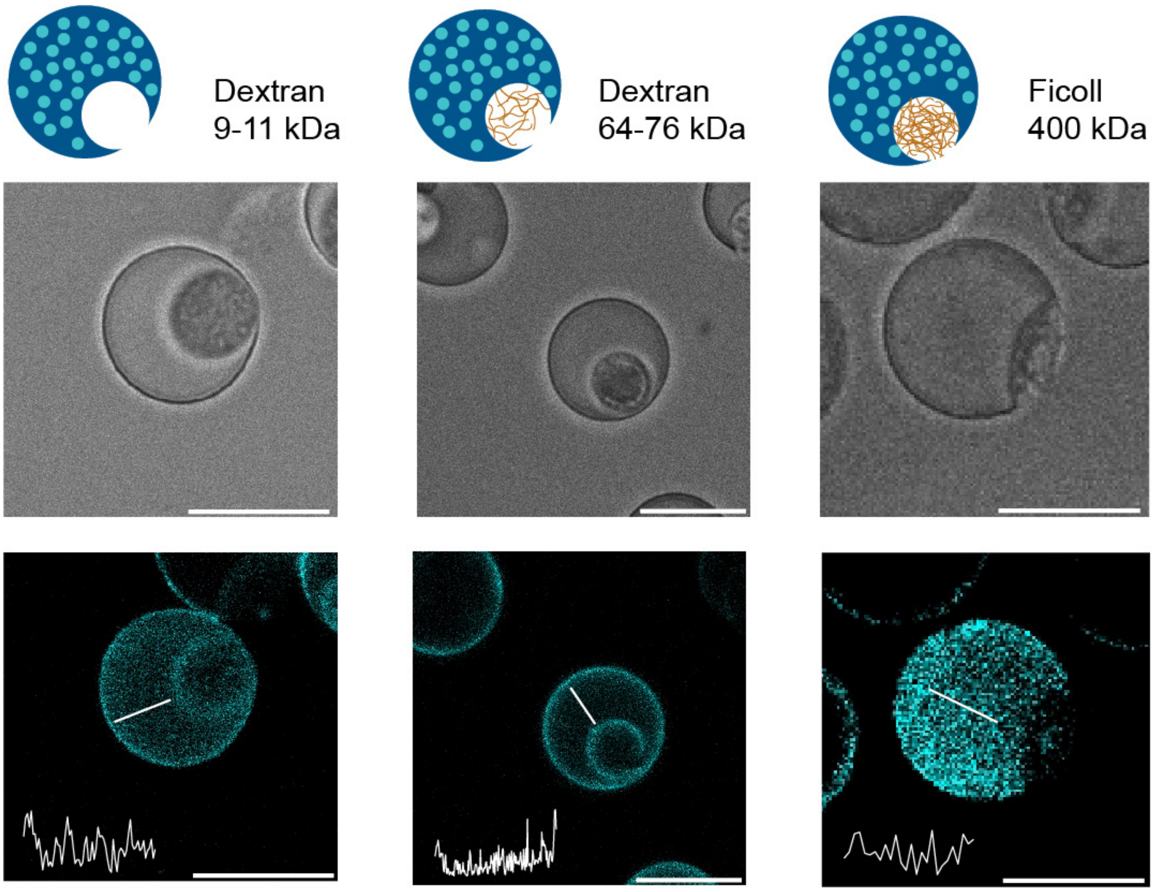


**Figure S4.** Confocal images of the fluorescently labelled catalase dissolved in the PEGDA phase. For all three polysaccharide systems a homogeneous distribution is obtained, as was expected. Scale bar is 20 μm.

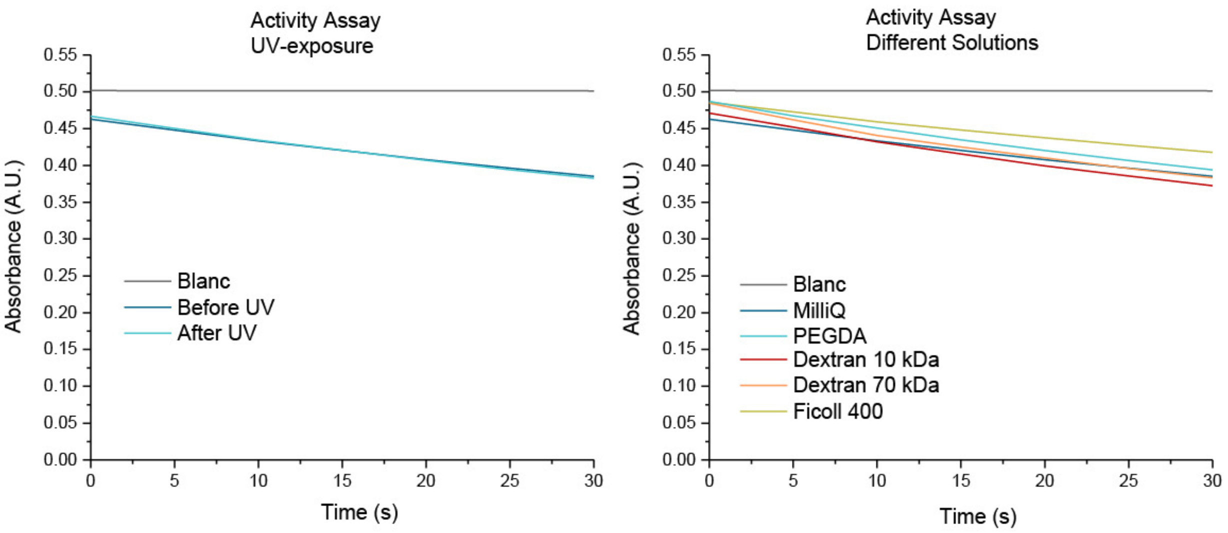


**Figure S5.** Activity assay of catalase before and after UV-exposure (left) and in different aqueous solutions (right). The UV-absorption of hydrogen peroxide was measured at a wavelength of 240 nm over time. Catalase decomposes hydrogen peroxide, this will decrease the hydrogen peroxide concentration and thus the absorbance. The activity of the enzyme under different circumstances can thus be studied. Hydrogen peroxide solution without the addition of enzyme was used as a blanc. The concentration remains constant and a horizontal trend is observed. The time dependent absorbance of hydrogen peroxide was measured for catalase before and after UV-exposure. As can be seen in de graph on the left, both lines overlap, from this we can conclude that the enzyme was not damaged by UV-exposure. In the graph on the right we plotted the activity assays of catalase in the different solutions of our micromotor system, including Milli-Q as a reference point. Here we observe that PEGDA, dextran 10 kDa and dextran 70 kDa have similar slopes which decrease faster than that of Milli-Q. Therefore, the enzyme is more active in these three solution compared to Milli-Q. Ficoll 400 on the other hand has a more shallow slope and catalase is thus less active.
